# Protection and Disinfection Activities of Oregano and Thyme Essential Oils Encapsulated in Poly(ε-caprolactone) Nanocapsules

**DOI:** 10.3390/molecules28031018

**Published:** 2023-01-19

**Authors:** Monika Hofbauerová, Magdaléna Rusková, Andrea Puškárová, Mária Bučková, Adriana Annušová, Eva Majková, Peter Šiffalovič, Giuseppe Granata, Edoardo Napoli, Corrada Geraci, Domenico Pangallo

**Affiliations:** 1 Institute of Physics, Slovak Academy of Sciences, Dúbravská Cesta 9, SK-84511 Bratislava, Slovakia; 2 Center for Advanced Materials Application, Slovak Academy of Sciences, Dúbravská Cesta 9, SK-84511 Bratislava, Slovakia; 3 Institute of Molecular Biology, Slovak Academy of Sciences, Dúbravská Cesta 21, SK-84551 Bratislava, Slovakia; 4 Istituto Chimica Biomolecolare-Consiglio Nazionale Delle Ricerche, Via Paolo Gaifami 18, 95126 Catania, Italy

**Keywords:** essential oils, poly(ε-caprolactone) nanocapsules, antimicrobial effect, reflectivity ratio, disinfection, environmental biofilm

## Abstract

The biocolonization of building materials by microorganisms is one of the main causes of their degradation. Fungi and bacteria products can have an undesirable impact on human health. The protection and disinfection of sandstone and wood materials are of great interest. In this study, we evaluated the protection and disinfection activity of oregano and thyme essential oils encapsulated in poly(ε-caprolactone) nanocapsules (Or-NCs, Th-NCs) against four types of environmental microorganisms: *Pleurotus eryngii*, *Purpureocillium lilacinum* (fungal strains), *Pseudomonas vancouverensis,* and *Flavobacterium* sp. (bacterial strains). The surfaces of sandstone and whitewood samples were inoculated with these microorganisms before or after applying Or-NCs and Th-NCs. The concentration-dependent effect of Or-NCs and Th-NCs on biofilm viability was determined by the MTT reduction assay. The results showed that Or-NCs and Th-NCs possess effective disinfection and anti-biofilm activity. Diffuse reflectivity measurements revealed no visible color changes of the materials after the application of the nanoencapsulated essential oils.

## 1. Introduction

Biodeterioration is one of the main causes of natural building material degradation [1]. Protecting these indoor and outdoor materials from biological contaminants is a challenging task. Under appropriate conditions that support microbial growth and proliferation such as high relative humidity and high temperature, fungi and bacteria are able to significantly contaminate natural materials. This leads to different types of damage on these materials, ranging from aesthetic to mechanical and chemical deterioration [2,3,4,5]. Even more serious is the impact of biological contamination on health. In fact, the microbial production of harmful substances such as allergens, mycotoxins, etc. is often associated with direct and indirect health effects [6].

Stone and wood are among the most widespread natural building materials. For this reason, their protection against microbial damage is a topic of great interest.

The bacteria *Pseudomonas vancouverensis* and *Flavobacterium* sp. were isolated from a sandstone slab of the Maximilian’s Fountain in Bratislava (Slovakia). Members of these genera are associated with the biodeterioration of sandstone [7].

The fungal strain *Purpureocillium lilacinum* was also isolated from the same sample of the Maximilian’s Fountain. This fungus is frequently encountered in soils and is also used as a bio-pesticide [8]. *P. lilacinum* is known to release nematocidal metabolites such as serine proteases, acetic acid, small-chain fatty acids, and leucinostatins, which plays a vital role as key toxicity factor in controlling nematode populations in soil [9]. *P. lilacinum* cutaneous infection can be highly mortal, due to the fungus’s ability to sporulate in tissues [10].

Fungi from the *Pleurotus* genus are wood-decay fungi responsible for the decomposition of wood [10]. *P. eryngi* demonstrated nematicidal activity against the larvae and adults of different nematodes species, in addition to proteases and chitinases production [11].

Essential oils (EOs) are natural green candidates for the protection and disinfection of many materials, including stone and whitewood, without using aggressive and toxic substances [12,13]. EOs are composed of complex mixtures of substances with antimicrobial activity. Due to this multicomponent composition, they can be used as effective antibiofilm, antifungal, insecticidal, and antibacterial agents [14,15,16].

Unfortunately, EOs are characterized by low water solubility, high volatility, and light, heat, and oxygen sensitivity. In fact, different chemical reactions, such as dehydrogenation or oxidation, triggered either enzymatically or chemically, can cause the degradation of EOs constituents [17]. Nanoencapsulation is a well-established approach for preserving EOs [18]. Several articles have been dedicated to the encapsulation of plant-derived metabolites such as EOs [19,20,21,22,23]. Choi et al. [24] found that the encapsulation of eugenol into poly(ε-caprolactone) nanocapsules could improve its stability against light degradation. Another study showed increased heat resistance of jasmine EOs after encapsulation in arabic gum and gelatin nanocapsules [25]. Jummes et al. [26] developed *Cymbopogon martini* EOs-loaded poly(ε-caprolactone) nanocapsules with a small particle size (282 nm) and high encapsulation efficiency (99.54%). Similarly, rosemary EO was efficiently entrapped in poly(ε-caprolactone) nanoparticles with an average size of 220 nm, a zeta potential equal to −19.9 mV, and an encapsulation efficiency of 99% [27]. To protect EOs from degradation and increase their physicochemical stability, a nanotechnology approach can be effective.

In previous works, we prepared nanocapsules based on poly(ε-caprolactone), a biodegradable and biocompatible polymer. NCs were loaded with thyme and oregano EOs, which contain a high amount of bioactive phenols such as thymol and carvacrol. We showed that the prepared nanostructured systems have a higher biological activity than EOs as they are [2,28].

In the current study, we evaluated the antibiofilm activity of Or-NC and Th-NC against two bacterial and two fungal strains which contaminate widely used building natural materials such as sandstone and whitewood. Moreover, under laboratory conditions, samples of sandstone and whitewood were used to prove the protective and disinfecting effect of Or-NC and Th-NC against the selected strains. A study of reflectivity was also performed to verify the color change of the tested sample materials.

This is an innovative approach for the application of nanoencapsulated EOs as protective and disinfecting agents for building natural materials, as there are very few publications dealing with similar research [2].

## 2. Results and Discussion

### 2.1. Biofilm Inhibitory Activity

**Figure 1:**
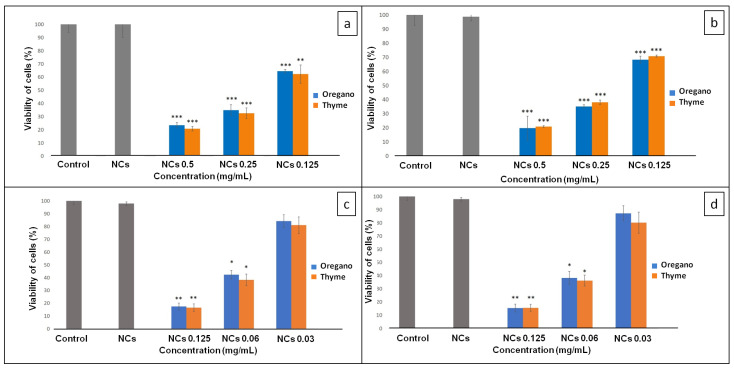
Viability of cells in the presence of encapsulated oregano and thyme EOs: (**a**) *P. vancouverensis*; (**b**) *Flavobacterium* sp.; (**c**) *P. eryngii*; (**d**) *P. lilacinum*. Where * is *p* < 0.05; ** is *p* < 0.01; *** is *p* < 0.001.

The inhibition of biofilm growth by EOs-NCs was confirmed at the tested MIC and at two sub-MIC concentrations (Table 1), i.e., 0.5, 0.25, 0.125 mg/mL for the bacterial strains (*P. vancouverensis*, *Flavobacterium* sp.) and 0.125, 0.06, 0.03 mg/mL for the fungal strains (*P. eryngii*; *P. lilacinum*) (Figure 1). As shown in Figure 1a,b, the tested concentrations of EOs-NCs against the bacterial strains resulted in a significant inhibition of biofilm formation (** *p* < 0.01, *** *p* < 0.001) compared to the control untreated sample (control sample without EOs-NCs). Similarly, Budri et al. [29] reported that the EOs of *Syzygium aromaticum* and *Cinnamomum zeylanicum* and their major components have a significant inhibitory effect on the biofilm production by *Staphylococcus aureus*.

However, no significant reduction was observed for the fungal strains with a concentration of 0.03 mg/mL (Figure 1c,d) compared to the control untreated sample. On the other hand, the EOs-NCs (0.06 mg/mL) reduced the viability of fungal cells significantly (* *p* < 0.05), and at a concentration of 0.125 mg/mL, a significant inhibition of biofilm formation (** *p* < 0.01) was observed. At the same time, the antimicrobial activity of empty NCs (without EOs) against all microbial cultures was also tested, and no antimicrobial effects were detected.

Sub-MICs of EOs-NCs (0.25 and 0.125 mg/mL) tested on *P. vancouverensis* showed similar results of biofilm inhibition for both concentrations of thyme (67.4% and 37.8%) and oregano (65% and 35.5%) EOs (Table 2). The efficiency of *Flavobacterium* sp. biofilm growth inhibition at sub-MICs of EOs-NCs was slightly lower, i.e., 61.9% and 29.1% for Th-NCs, and 64.8% and 31.6% for Or-NCs.

The sub-MICs for the fungal strains were lower in comparison with the concentrations used for inhibiting the growth of the bacterial strains. The efficiency of *P. eryngii* biofilm inhibition by 0.06 mg/mL of Th-NCs/Or-NCs was around 63%; however, for when using 0.03 mg/mL of EOs-NCs, there was a significant difference in inhibition efficiency between thyme and oregano (Table 2). The same results were observed for the *P. lilacinum* biofilm inhibition at both concentrations of EOs-NCs. Based on these results, we concluded that the thyme EO is more effective in inhibiting fungi growth than the oregano EO. These observations are also in agreement with previous studies. Abd El-Aziz et al. [30] demonstrated that chitosan nanoparticles containing mint EOs had an inhibitory effect against *Aspergillus niger*. Similarly, Izadi et al. [31] showed that chitosan nanoparticles loaded with *Carum copticum* EOs at a concentration of 200 ppm exerted a complete inhibitory effect on the pathogenic fungus *Alternaria alternata*.

### 2.2. Determination of Microbial Activity on Wood and Sandstone Surfaces

**Figure 2:**
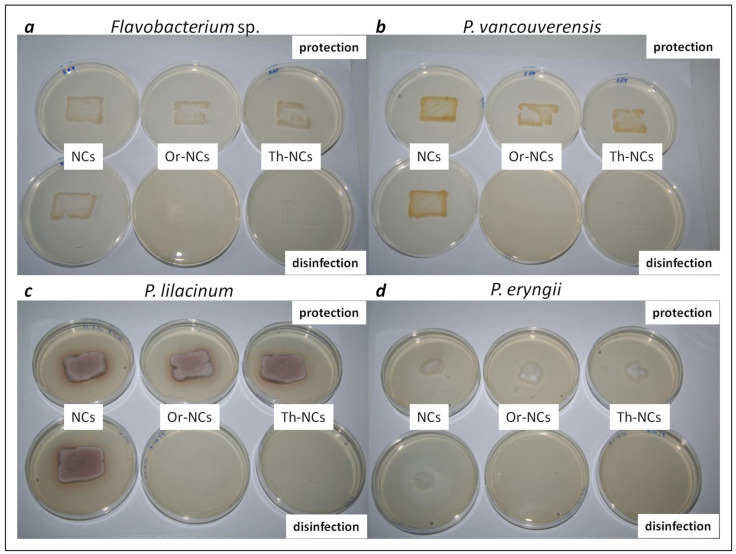
Microbial cultures transferred from the material surfaces treated with empty NCs and EOs-NCs (tested for their protection and disinfection effects) to Petri dishes, using the agar plate printing method. The pictures show bacteria and fungi biofilms, (**a**) *Flavobacterium* sp. (sandstone); (**b**) *P. vancouverensis* (sandstone); (**c**) *P. lilacinum* (sandstone); (**d**) *P. eryngii* (whitewood).

The microbial activity on material surfaces was monitored through an imprint of bacteria/fungal cells from a material surface into a Petri dish using the agar plate printing method (Figure 2). The antimicrobial activity of NCs without EOs and EOs-NCs was tested for all microbial cultures to evaluate the possibility to disinfect the contaminated surfaces and surfaces protection (safeguarding the surfaces against microbial colonization). The growth of bacterial/fungal cells after depositing 10^6^ colony-forming units (CFU)/mL above or below the NCs/EOs-NCs layers at concentrations corresponding to the MIC values (Table 1) was monitored. The bacterial cells *P. vancouverensis* and *Flavobacterium* sp. (examined to test the protection of sandstone) showed a higher log_10_ reduction in the number of colony-forming units (CFU) than the fungi *P. lilacinum* (examined to test the protection of sandstone) and *P. eryngii* (examined to test the protection of whitewood) (Table 3). Similar results were reported in our previous works [32] for *S. aureus*, with a MIC of 0.5 mg/mL. We achieved very interesting results when observing the disinfecting effect of EOs-NCs, as we detected a 100% antimicrobial effect for all samples. This effect was confirmed by measuring the number of colony-forming units (CFU) on agar plates and by digital optical microscopy (Table 3 and Table 4).

The areas covered by the microbial cells on the agar plates were determined by digital optical microscopy. The bacterial and fungal areas were calculated using ImageJ analyzing software by defining the microbial cells border. The results from the microbial cells area measurements are presented in Table 4. In terms of protection, the largest area of imprinted microbial cells was observed for *P. vancouverensis* treated with NCs, with a size of 793.1 mm^2^ (the area on sandstone was 600 mm^2^). This was a 32% larger area compared with the substrate area without treatment. Similar results were obtained for *Flavobacterium* sp. and *P. lilacinum*. Significant results were achieved on wood treated with Th-NCs, where the surface of the imprinted *P. eryngii* was only 244.4 mm^2^ (the wood area was 1600 mm^2^).

In terms of disinfection, the microbial activity of the bacterial and fungal cells was completely suppressed on surfaces treated with EOs-NCs. These unequivocal results prove the possibility of using encapsulated EOs for the disinfection of various surfaces. There are few studies on the role of nanoencapsulated EOs for preventing the biodegradation of wood [33,34] and stone [2,35]. The effect of reducing bacterial or fungal biofilms on the surface of different materials by EOs has been successfully demonstrated in several studies [36,37,38,39,40,41,42].

### 2.3. Measurement of the Optical Properties of Materials before and after the Application of NCs and EO-NCs in the Presence or Absence of Microbial Cultures 

EOs-NCs should protect materials against microbial damage, but they should also preserve the physical material properties, such as appearance and color. The measurement of the optical properties using integral surface reflectivity, which includes diffuse and specular reflectivity components, is a direct method of determining the color change of a material after applying a protective layer. This method consists in measuring first the reflectivity of a clean substrate (without a coating of EOs-NCs) and subsequently its reflectivity after the deposition of EOs-NCs. To determine the spectral reflectivity changes after the protective layer application, we used the reflectivity ratio parameter R_r_ in the visible spectral region (400–700 nm), according to the equation [12]:

R_r_ = R _(coating)_/R _(substrate)_ (1)

where R_(coating)_ is the reflectivity after the application of the NCs and EOs-NCs layer on the substrate, and R_(substrate)_ is the reflectivity of the clean substrate.

**Figure 3:**
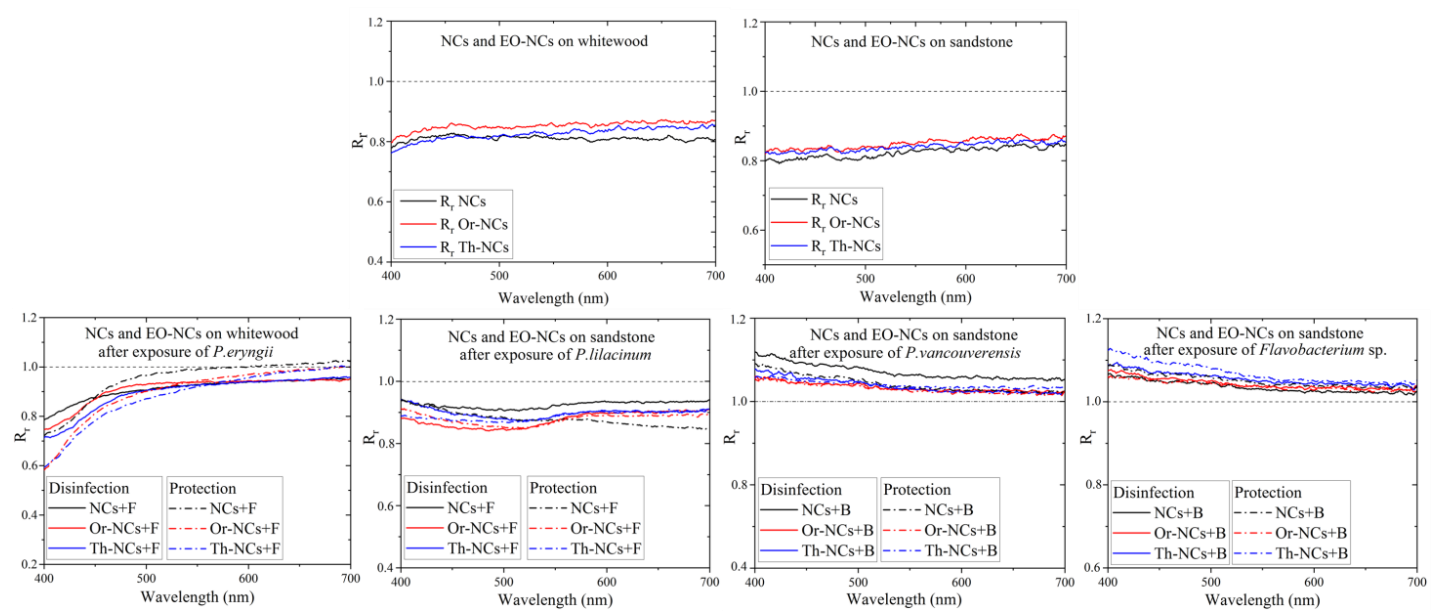
Reflectivity ratio R_r_ as a function of the wavelength for whitewood and sandstone samples before and after the application of microbial cells.

Figure 3 shows the R_r_ values of the whitewood and sandstone substrates. The R_r_ changes due to the application of NCs and EO-NCs on wood and sandstone before microbial cells exposure in the visible region were very similar. The general trend for both surfaces with the applied NCs and EOs-NCs was a decreased substrate reflectivity in the visible spectrum due to a substantial absorption cross-section of EOs and NCs in the visible spectral region. The reflectivity changed significantly after exposure to the microbial cultures. A significant shift in R_r_ close to 1 was detected after adding *P. eryngii*, while the reflectivity after exposure to *P. lilacium* was approximately the same as before the exposure. On the other hand, after exposure of the bacterial cells to a substrate, we observed a significant reflectivity increase in the blue-green part of the visible spectrum. These results suggested that the reflectivity measurement is a suitable method for detecting sandstone deterioration by bacterial cells because it allows measuring a significant shift in the R_r_ values in the visible spectral region.

**Figure 4:**
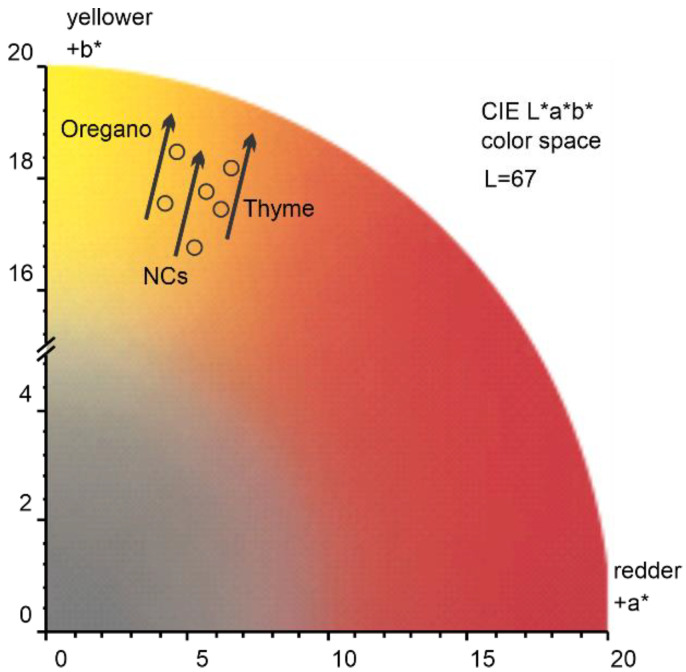
Sandstone color changes after the application of Or-NCs, Th-NCs, and NCs, encoded in CIE L*a*b* color space.

To account for a perceived color change after the application of Or-NCs, Th-NCs, and NCs, we evaluated the color changes in CIELAB color space (CIE L*a*b*), which was defined by the International Commission on Illumination (abbreviated CIE) in 1976. A CIE standard illuminant D65 (T = 6500 K) was used to calculate the a* and b* values. As a representative example, Figure 4 shows the color change of sandstone after the application of Or-NCs, Th-NCs, and NCs. The effect of all applied films resulted in a similar overall color change of $\Delta E_{ab}^{*}$ = 1 ± 0.13, with b* being the major component, underlying a perceived color change toward a yellow hue indicated by a black arrow in Figure 4.

To summarize, the total change in diffuse reflectivity after applying NCs and EOs-NCs as a protective layer on a clean substrate was about 17%, meaning that the color change after applying the protective layer to the material surface was challenging to see with the naked eye.

**Figure 5:**
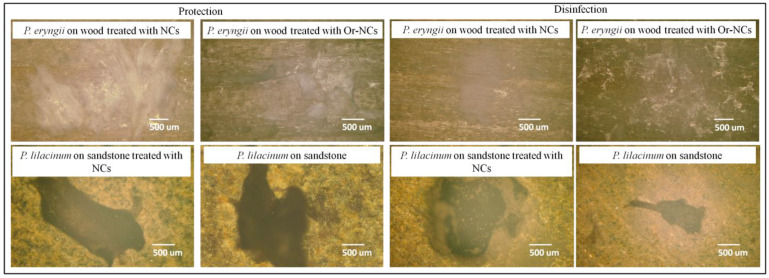
*P. eryngii* and *P. lilacinum* on wood and sandstone observed by digital microscopy.

The direct method of detecting microbial cells on a substrate surface, such as digital microscopy, is complex due to the porosity and roughness of the treated material. Bacterial cells on the sandstone surface could not be observed. The ability of fungal cells to form hyphae and spores allows a more straightforward penetration into the structure of materials and growth on the material surface. Figure 5 represents fungal cells of *P. eryngii* and *P. lilacinum* on a wood or sandstone surface untreated and treated with NCs and Or-NCs analyzed by digital microscopy. After exposure to *P. eryngii*, white fungal spores were visible on wood. This observation is consistent with the diffuse reflectivity measurement, where the reflectivity ratio R_r_ of the individual samples (disinfection and protection effect) was close to 1 after exposure to P. *eryngii*. It means that the reflectivity after exposure to fungal cells was higher than for samples that were not exposed to the fungal cells. In this case, the reflectivity measurement could be a suitable method for detecting fungal cells on wood surfaces.

In contrast, this effect was not observed for sandstone exposed to *P. lilacinum*. The dark spots of fungal contamination on the surface led to higher R_r_ values, rather than to lower values (even if only minimally). This shift in reflectivity can be attributed to the larger porosity and roughness of sandstone.

## 3. Materials and Methods

### 3.1. Nanocapsules and Essential Oils

Poly(ε-caprolactone) (PCL) nanocapsules loaded with oregano and thyme EOs were prepared according to the procedure described by Granata et al. [28] using the interfacial deposition of the preformed polymer method. Briefly, an acetone solution (80 mL) (Sigma-Aldrich/Merck, Darmstadt, Germany) containing EO (1.0 g), PCL (320 mg), and sorbitan monostearate (112 mg) (Sigma-Aldrich/Merck) was dropped under stirring at 25 °C into a polysorbate 80 aqueous solution (275 mL in 160 mL) (Sigma-Aldrich/Merck). The removal of the organic solvent provided an EO-NC suspension. The NCs without essential oils were prepared in the same conditions. The physicochemical parameters of the nanocapsules such as particle size, polydispersity, and zeta potential, were determined by light scattering measurements. The encapsulation efficiency and loading capacity values were obtained by UV–Vis spectroscopy. The exact composition of the commercial thyme and oregano EOs (provided by Flora s.r.l., Lorenzana, Italy, and Esperia S.p.A, Milan, Italy) were determined by GC-FID and GC-MS analysis. The values allowing the characterization of the nanocapsules and EOs were already reported [10] and are briefly shown in Table 5 and Table 6.

### 3.2. Microbial Strains and Growth Conditions

The environmental bacterial strains *Pseudomonas vancouverensis* and *Flavobacterium* sp. and the fungal strain *Purpureocillium lilacinum* were isolated from a sandstone slab (Maximilian’s Fountain in Bratislava, Slovakia) and belong to the collection of the Institute of Molecular Biology Slovak Academy of Sciences (IMB SAS). *Pleurotus eryngii* CCBAS471, was obtained from the Culture collection of Basidiomycetes (CCBAS), Institute of Microbiology, Academy of Sciences of the Czech Republic. The bacterial strains were kept frozen in stock cultures at −80 °C, and the fungal cultures were stored at 4 °C and subcultured once a month. The bacteria were grown at 26 °C in nutrient agar (NBA) (HiMedia, Mumbai, India) for 12–18 h. The fungal strains were grown at 26 °C on Malt Extract Agar (MEA) (HiMedia) for 5 days.

### 3.3. Minimum Inhibitory Concentration (MIC)

Microtiter plate assays were performed according to Poaty et al. (2015) [43] with a modification to determine the MIC (minimum inhibitory concentrations) of Th-NCs and Or-NCs, inhibiting bacterial and fungal growth [10]. The MIC for the fungal strains was determined in our previous work [32]. We based our testing of anti-biofilm activity on BIC concentrations determined by Kapustova et al. 2021 [10].

### 3.4. Microtiter Biofilm Assay

Biofilm formation was processed as described by Harriot et al. [44], with minor modifications. The bacterial cultures (*P. vancouverensis*, *Flavobacterium* sp.) were grown overnight in nutrient broth and then were washed twice in sterile phosphate-buffered saline (PBS) (Sigma-Aldrich/Merck) by centrifugation at 3000× *g* for 5 min at room temperature. The obtained bacterial cell suspensions were diluted to a concentration of 2 × 10^5^ CFU/mL. The isolates of fungal spores of *P. lilacinum* and *P. eryngii* were harvested from a 5-day-aged pure culture in MEA by adding 5 mL of saline solution (0.85%) to the plate. The spore suspensions were resuspended by vortexing before quantification. The final pellets of fungal cells were dissolved in approximately 20 mL of Malt Extract Broth (MEB; HiMedia) and counted under a microscope; the final density of the fungal cell suspensions was 2 × 10^5^ spores/mL.

The microtiter biofilm assay took place in 96-well microtiter plates. The EOs-NCs testing proceeded as follows: 100 µL of bacterial or fungal cell suspensions, 90 µL of nutrient broth (NB; HiMedia) or MEB, and 10 µL of EOs-NCs in various concentrations were added to the plate wells. The original concentrations of EOs in NCs was 5.7 mg/mL for the thyme EO and 5.8 mg/mL for the oregano EO. These concentrations were adjusted with respect to the MIC and sub-MIC values for bacterial and fungal cell suspensions from 0.5 to 0.125 mg/mL and from 0.125 to 0.03 mg/mL, respectively. The final volume in each well was 200 µL.

Each bacteria plate was statically incubated for 24 h at 26 °C. After incubation, the supernatant was removed, and each well was rinsed 2 times with 90 µL of sterile PBS. Subsequently, 10 µL of 3-(4,5-dimethyl-thiazoyl)-2,5-diphenyltetrazolium bromide (MTT) (5 mg/mL) (Sigma-Aldrich/Merck) was added to the plate, which was incubated again for 2 h at 26 °C. After incubation, 100 µL of detergent (mixture of 95% isopropyl alcohol and 2M HCl) (CentralChem, Bratislava, Slovakia) was added and mixed, and the biofilm was characterized by optical density measurements. The measurements took place at 540 nm using an 800™ TS Absorbance Reader (BioTek, Winooski, VT, USA). With the measured absorbance values, we determined the MIC and sub-MIC.

Each fungal plate was incubated statically for 48 h at 26 °C. After biofilm formation, the MEB medium was carefully removed, and the fungal biofilm was quantified with 0.1% crystal violet solution (CV) as follows: 110 μL of 0.1% CV was pipetted into each well of dried biofilm and left for 5 min at room temperature. Then, the CV solution was withdrawn, and the wells washed twice with 100 μL of PBS and dried for 15 min at room temperature. Next, we added 200 μL of 95% ethanol into each well and left it for 15 min to release CV from the cells. After this time, 150 μL of inoculum was transferred to a clean 96-well microtiter plate, and we determined the MIC and sub-MIC values by optical density measurements at 570 nm by an 800™ TS Absorbance Reader (BioTek) [45].

The control samples of biofilm formation from bacterial and fungal cell suspensions without EOs-NCs consisted of 100 µL of cells and 100 µL of medium per one well.

The data are presented as means of 3 experiments ± one standard deviation (SD). The differences between the groups were tested for statistical significance using the Student’s t-test (* *p* < 0.05; ** *p* < 0.01; *** *p* < 0.001). Because the antimicrobial activity datasets were normally distributed, the independent samples t-test was performed to test for significant differences between groups.

### 3.5. Antimicrobial Activity on the Sandstone and Whitewood Samples

Sandstone and whitewood were cut into rectangular units with a size of approximately 30 × 20 × 10 mm (L × W × D) for sandstone and of 40 × 40 × 10 mm (L × W × D) for whitewood. Stone and whitewood were sterilized by autoclave (121 °C for 20 min) and kept under a laminar flow for about 1 h. Two methods were tested and compared: disinfection (for disinfecting contaminated surfaces) and protection (for safeguarding surfaces against microbial colonization).

Disinfection procedure: 100 μL of bacterial/fungal suspension (1 × 10^6^ CFU/mL) was spread on the surface of each sample. After absorption/evaporation of the bacterial/fungal suspension, 200 μL of Th-NCs and Or-NCs (with a concentration of EOs of 0.5 mg/mL for the bacterial surfaces and of 0.125 mg/mL for the fungal surfaces) was added to the sandstone and whitewood surfaces.

Protection procedure: 200 μL of Th-NCs and Or-NCs (with a concentration of EOs of 0.5 mg/mL for the bacterial surfaces and 0.125 mg/mL for the fungal surfaces) was added to the sandstone and whitewood surfaces. After drying the surfaces, 100 μL of bacterial/fungal suspension (1 × 10^6^ CFU/mL) was added to the sandstone and whitewood surfaces.

The two bacterial strains (*P. vancouverensis* and *Flavobacterium* sp.) and the fungus *P. lilacinum* were inoculated on the sandstone samples, while *P. eryngii* was applied on the whitewood surface. After applying the microbial suspensions and EOs-NCs to the material surfaces, the tested materials were dried at room temperature. The treated surfaces were printed on MEA or NBA in order to transfer the microorganisms from sandstone and whitewood to agar medium via direct contact for 15 min. The conditions were standardized by applying 0.02 kg/cm^2^ of constant pressure. This ensured the absence of air bubbles and perfect adhesion of the agar to the sandstone and whitewood surfaces [2]. The agar plates were incubated at 26 °C for 48 h (bacteria) and at 26 °C for 96 h (fungi), and colony counts were carried out. The test was performed three times, and the average colony count of duplicate printed plates was used to calculate the CFU/mL.

### 3.6. Detection of Microbial Activity on the Substrates’ Surface

The printed biofilms on Petri dishes (mm^2^) were evaluated by using ImageJ program. A digital optical microscope from Keyence (Osaka, Japan) with a long working distance zoom objective was used to display the grown microbial culture on the wood and sandstone surfaces.

### 3.7. Measurement of Optical Properties 

The reflectivity measurements were used to determine the optical properties of whitewood and sandstone with empty NCs and EOs-NCs before and after exposure to the microorganisms. The measurements were performed using a SolidSpec-3700 UV–VIS-NIR spectrophotometer from Shimadzu (Kyoto, Japan). The diffuse reflectivity measurements were recorded in the range from 200 to 900 nm using an integrating sphere equipped with three detectors: a photomultiplier, InGaAs, and PbS. In addition, the baseline correction prior to the reflectivity measurement was performed using a standard white plate composed of BaSO_4_.

## 4. Conclusions

The goal of our work was the discovery of potential candidates for the protection and disinfection of building materials against bacterial and fungal strains isolated from whitewood and sandstone, responsible for the deterioration of these materials. We used oregano and thyme essential oils encapsulated in polymeric nanocapsules, which possess greater biological activity than free essential oils. These biodegradable and biocompatible nanosystems have been shown to act as potential disinfectant agents on whitewood and sandstone samples inoculated with bacterial or fungal strains. Furthermore, the thyme and oregano nanocapsules were able to inhibit the formation of biofilm by the selected microorganisms. For these reasons, these green nanosystems could potentially be useful to disinfect whitewood and sandstone materials from microbial biocolonization, consequently avoiding any undesirable impact on human health.

## Figures and Tables

**Table 1:** MIC and sub-MIC values of EO-NCs against *P. vancouverensis*, *Flavobacterium* sp., *P. eringii*, *P. lilacinum*.

Strain	MIC (mg/mL)	Sub-MIC (mg/mL)	
	Th-NCs/Or-NCs	Th-NCs/Or-NCs	
*P. vancouverensis*	0.5	0.25	0.125
*Flavobacterium* sp.	0.5	0.25	0.125
*P. eringii*	0.125 *	0.06	0.03
*P. lilacinum*	0.125 *	0.06	0.03

* [10].

**Table 2:** Biofilm growth inhibition efficiency of EO-NCs against *P. vancouverensis*, *Flavobacterium* sp., *P. eringii, P. lilacinum*.

Strain	Sub-MIC/Biofilm Growth Inhibition Efficiency (%)			
	Th-NCs		Or-NCs	
*P. vancouverensis*	0.25 (67.4%)	0.125 (37.8%)	0.25 (65%)	0.125 (35.5%)
*Flavobacterium* sp.	0.25 (61.9%)	0.125 (29.1%)	0.25 (64.8%)	0.125 (31.6%)
*P. eringii*	0.06 (63.7%)	0.03 (19.8%)	0.06 (61.75%)	0.03 (12.63%)
*P. lilacinum*	0.06 (63.7%)	0.03 (19.8%)	0.06 (61.7%)	0.03 (12.7%)

**Table 3:** Concentration of microorganisms (log_10_ CFU/mL) grown on printed agar plates.

	*P. vancouverensis*	*Flavobacterium* sp.	*P. lilacinum*	*P. eryngii*
	Sandstone	Sandstone	Sandstone	Whitewood
Protection	log_10_ CFU/mL			
NCs	7.10	7.13	7.21	7.24
Or-NCs	4.04	4.06	5.10	5.14
Th-NCs	4.05	4.08	5.13	5.16
	*P. vancouverensis*	*Flavobacterium* sp.	*P. lilacinum*	*P. eryngii*
Disinfection	log_10_ CFU/mL			
NCs	6.05	6.06	6.13	6.11
Or-NCs	ND	ND	ND	ND
Th-NCs	ND	ND	ND	ND

Th-NCs and Or-NCs (0.5 mg/mL of essential oil for bacterial surfaces and 0.125 mg/mL for fungal surfaces); ND, not detected.

**Table 4:** Measurement of microbial cells areas on Petri dishes.

Protection	*P. vancouverensis*	*Flavobacterium* sp.	*P. lilacinum*	*P. eryngii*
	Sandstone	Sandstone	Sandstone	Whitewood
NCs	793.1 mm^2^	736.0 mm^2^	603.0 mm^2^	287.7 mm^2^
Or-NCs	553.6 mm^2^	486.5 mm^2^	535.9 mm^2^	416.0 mm^2^
Th-NCs	618.1 mm^2^	449.4 mm^2^	619.3 mm^2^	244.4 mm^2^
Disinfection	*P. vancouverensis*	*Flavobacterium* sp.	*P. lilacinum*	*P. eryngii*
NCs	675.2 mm^2^	681.5 mm^2^	542.3 mm^2^	192.2 mm^2^
Or-NCs	0 mm^2^	0 mm^2^	0 mm^2^	0 mm^2^
Th-NCs	0 mm^2^	0 mm^2^	0 mm^2^	0 mm^2^

**Table 5:** Physicochemical characterization of EO-NCs and NCs (empty).

	*Z* -Average Diameter (nm)	PDI	ζ (mV)	EE%	LC%
NCs	185 ± 1	0.10 ± 0.03	−13 ± 1	-	-
Th-NCs	198 ± 3	0.09 ± 0.02	−11 ± 1	84 ± 6	52 ± 3
Or-NCs	200 ± 3	0.05 ± 0.03	−10 ± 2	80 ± 9	51 ± 4

PDI, polydispersity index; ζ (mV), zeta potential; EE%, encapsulation efficiency; LC%, loading capacity.

**Table 6:** Chemical composition of commercial *Origanum vulgare* and *Thymus capitatus* essential oils.

RI lit ^a^	RI exp ^b^	Class/Compound ^c^	*O. vulgare* % ^d^	*T. capitatus* % ^d^
		**Monoterpene hydrocarbons**	**27.99**	**22.26**
939	931	α-Pinene	1.37	0.79
991	986	β-Myrcene	0.66	1.29
1017	1013	α-Terpinene	0.36	1.03
1025	1027	*p*-Cymene	21.54	9.26
1060	1058	γ-Terpinene	2.16	8.22
		**Oxygenated monoterpenes**	**68.66**	**72.88**
1097	1098	Linalool	4.26	0.86
1290	1303	Thymol	25.02	0.58
1299	1319	Carvacrol	35.95	69.91
		**Sesquiterpenes**	**1.94**	**3.19**
1419	1425	β-Caryophyllene	1.70	2.56
		**Others**	**0.47**	**0.22**
		**TOTAL**	**99.06**	**98.55**

^a^ Literature retention index (RI); ^b^ Retention index (RI) relative to standard mixture of *n*-alkanes on an SPB-5 column; ^c^ Identified compounds (<1.00% in both samples have not been reported, a complete compounds list has been previously reported: Kapustova et al., 2021) [10]; ^d^ Relative peak area percent represents the average of 3 determinations.

## Data Availability

Not applicable.

## Sample Availability

Samples of the compounds are available from the authors.
